# 

**Published:** 1861-05

**Authors:** 


### CHICAGO MEDICAL JOURNAL.

**Terms, $2.00 per Annum.**

### CONTENTS OF THE MAY NUMBER.

ORIGINAL COMMUNICATIONS.

PAGE.

Rare Case of Spina Bifida. By Chas. M. Anderson, M.D., Minneapolis, Minn. 257
Treatment of Ununited Fractures. By Dr. J. C. Stone.................. 259
Removal of Parotid Tumor. By Dr. R. L. Rea, M. D., Prof. Anat., Rush Medical College............................................. 260
DeWitt County Medical Society........................................ 264

EDITORIAL.

Editorial and Miscellaneous.......................................... 268
Subcutaneous Osteotomy.—Report of a Case of Anchylosis of the Knee Joint Successfully Treated by Subcutaneous Division of the Condyles of the Femur, with Observations on the Application of this Method of Treatment. By Daniel Brainard, M. D., Professor of Surgery in Rush Medical College.................................. 297
Editorial and Miscellaneous.......................................... 308

SELECTED.

Alterations of the Tongue in Diseases. By Dr. Carl Neidhardt........... 311
True Ringworm. By Jona. Hutchison, of the London and Metropolitan Hospitals.—Extract........................................ 314
A Sensible Judge's Charge to the Jury in a Suit for Malpractice......... 315

